# Supplementary material for: All-optical control of second-harmonic generation in β-BaB2O4 via coherent, terahertz-driven acentric lattice displacement
Source: Nat Commun. 2026 Apr 7;17:4938. doi: 10.1038/s41467-026-70532-x (PMC13234348; doi:10.1038/s41467-026-70532-x)
Supplement: Supplementary file 1 — Supplementary Information [file 41467_2026_70532_MOESM1_ESM.pdf]

Supplementary Information: All-optical control of second-harmonic generation in β-BaB_2O_4 via coherent, terahertz-driven acentric lattice displacement

Flavio Giorgianni[1,2,*], Nicola Colonna[2], Gabriel Nagamine[1], Leonie Spitz[2], Guy Matmon[2], Alexandre Trisorio[2], Nicolas Forget[3], Carlo Vicario[2], Adrian L. Cavalieri[1,2]

[1]Institute of Applied Physics, University of Bern, CH-3012 Bern, Switzerland
[2]Paul Scherrer Institute, CH-5232 Villigen-PSI, Switzerland
[3]Institut de Physique de Nice (INPHYNI), Université Côte d'Azur

1. Experimental Setup and THz Field Characterization

A schematic of the experimental geometry is shown in Supplementary Fig. 1a. The measurements are made using a 100 Hz, 20 mJ, 50 fs Ti:Sa laser system coupled to an optical parametric amplifier (OPA). The output energy of the OPA signal beam was approx. 3.9 mJ per pulse. The OPA signal, at a wavelength of 1.5 µm, was employed to generate intense single-cycle THz pulses through optical rectification in a large-aperture DAST crystal, with clear aperture exceeding 10 mm in diameter. The OPA signal beam diameter was approximately 9 mm at the crystal surface. Metallic multi-mesh filters were inserted into the THz optical path to isolate the THz pulses from the residual OPA beam and to shape the THz pulse spectrum.

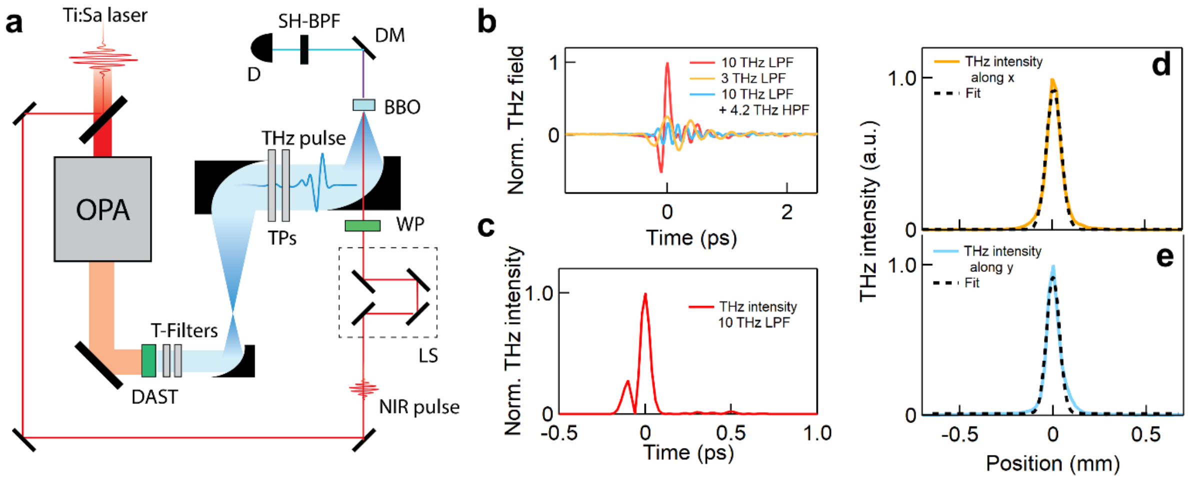

***Supplementary Fig. 1. Experimental Setup and THz field characterization. a**, Setup for THz generation and modulation of second harmonic in BBO. LS: linear stage; WP: half-wave plate, DM, dichroic mirror, SH-BPF: band-pass filter for SH signal, T-Filter: THz spectral filters, TPs: THz polarizers, BBO: BBO crystal sample. **b**, THz waveforms for 10 THz LPF, 3 THz LPF, and 4.2 THz HPF+10 THz LPF. **c**, Intensity profile $|E(t)|^2$ of the THz pulse with the 10 THz LPF. **d**, Horizontal and **e**, vertical THz beam transverse profile of THz pulse at the sample position with the 10 THz LPF. Orange and light blue solid curves are the experimental data measured with a THz camera while black dotted lines represent a Gaussian fit. The estimated FWHM are 90 µm and 85 µm,* respectively.

The THz beam was expanded and then focused onto the β-BaB_2O_4 (BBO) crystal sample using three off-axis parabolic mirrors with respective focal lengths of 1", 6", and 2" (see Supplementary Fig. 1a), which permits tight focusing of the THz beam onto the sample. A pair of wire-grid THz polarizers are used to vary the THz electric field strength at the sample (TPs in Supplementary Fig. 1a). The rotation

angle of first polarizer is adjustable, while the second polarizer was fixed to transmit only the vertically polarized component.

For the broadband THz-pump experiments (see Fig. 1c–f in the main text), isolation of the OPA signal beam was achieved using a filter set consisting of two 20 THz low-pass filters (LPFs) and one 10 THz LPF (all from QMC Ltd). This configuration provided a measured extinction ratio of $\sim 10^7$ for the residual 1.5 μm OPA pulse. To selectively tailor the THz pump spectrum relative to the phonon resonance (see Fig. 2a of the main manuscript), the 10 THz LPF in the filter set was replaced as follows: (i) for isolating frequency components below the phonon resonance, a 3 THz LPF was used; (ii) for isolating components above the resonance, a combination of a 4.2 THz high-pass filter (HPF) followed by a 10 THz LPF was used. The temporal profile of the THz pulse at the sample position was measured using electro-optic sampling (EOS) with a (110) GaP crystal of 200 μm thickness. The THz waveforms measured by EOS for these three filter configurations are shown in Supplementary Fig. 1b.

The broadband THz electric field strength was estimated from measurement of the temporal profile of the THz pump, the THz pulse energy, and the focused THz spot size. The intensity temporal profile of the broadband pump pulse computed as the square of the THz electric field waveform is shown in Supplementary Fig. 1c. The transverse spatial profile of the THz beam was measured using a THz micro-bolometric camera (NEC IRVT0830). The THz energy per pulse was measured with a Gentec THZ12D-3S-VP-D0 energy meter.

The THz pulse in Fig. 1 of the main manuscript has the following parameters: THz energy/pulse: 3.5 μJ, focused spotsize (FWHM): 90 μm from Gaussian fit of the beam profile (see Supplementary Fig. 1d-e).

A THz field strength, $E_{THz} \approx 8.5\ MV/cm$, was estimated using the following expression[1]:

$$E_{THz} = \sqrt{\frac{z_0 W}{\pi w^2 \int_{-\infty}^{\infty} g^2(t)dt}} \quad \text{(S1)}$$

where $z_0 = 377\ \Omega$ is the free space impedance, W is the THz energy/pulse, w is the beam waist and $g^2(t)$ is the temporal intensity profile of the THz pulse with peak value normalized to 1, (see Supplementary Fig. 1b). The temporal profile $g(t)$ was measured by EOS and the integral, $\int_{-\infty}^{\infty} g^2(t)dt$ = 0.086 ps. The THz field strengths for pump spectra above and below the phonon resonance were determined from EOS measurements. The THz pulse below the phonon resonance (obtained using the 3THz LPF), has a peak electric field that is 4 times smaller than the peak electric field of the broadband THz pulse obtained with the 10 THz LPF alone (see Supplementary Fig. 1b). Above resonance (implemented using the 4.2 THz HPF in combination with the 10 THz LPF), the peak THz field amplitude was 0.16 times the peak field of the broadband THz pump pulse.

2. Evidence of THz-field-induced second harmonic intensity modulation in BBO

To verify that the observed SH intensity modulation does not result from variations in the intensity of the transmitted fundamental frequency (FF) beam potentially due to THz-induced changes in the Fresnel coefficients, the transmitted NIR pulse intensity was measured as a function of the time delay relative to the THz pump pulse (see Supplementary Fig. 2). No significant modulation is observed in the NIR pulse intensity, confirming that the SHG modulation originates from the resonant THz-driven phonon excitation.

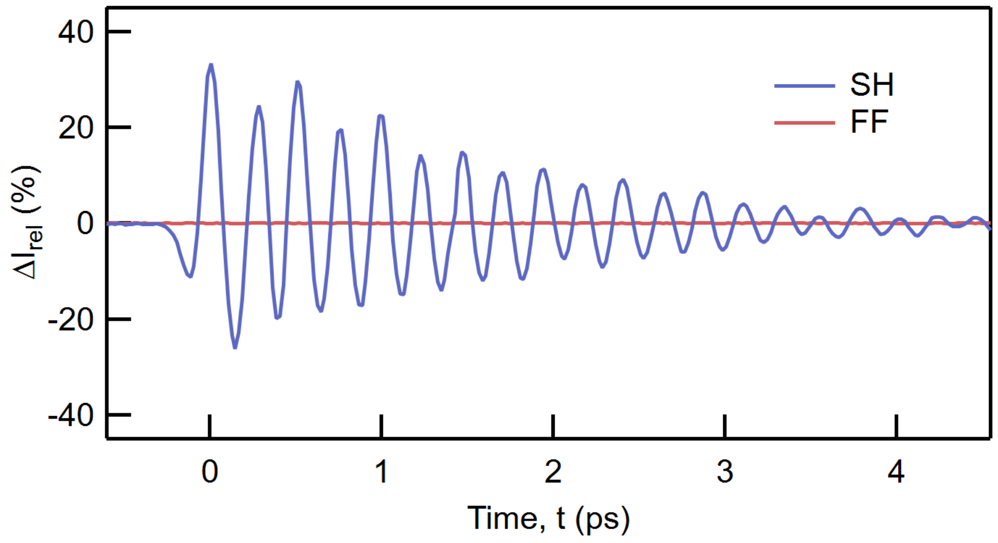

Supplementary Fig. 2. Experimental measurement of THz-field-induced modulation of SH and FF intensities. *The absence of significant modulation in the FF beam intensity confirms that the observed modulation effect originates from the SH process.*

3. THz Spectroscopy of BBO and Laue X-ray Diffraction

To characterize the BBO infrared-active phonons, optical transmission measurements in the THz regime were performed using Fourier transform infrared (FTIR) spectroscopy at the IR beam line of the Swiss Light Source at PSI. For these measurements, a 30-µm-thick reference BBO crystal was used with the same orientation ($\theta = 29.3°$ and $\phi = 90°$) as the crystal in the main THz experiment, but with reduced thickness to maximize the THz transmission. Supplementary Fig. 3 shows the transmission spectra in the THz range for light polarised along the ordinary-axis, which is consistent with previously reported work[2–4].

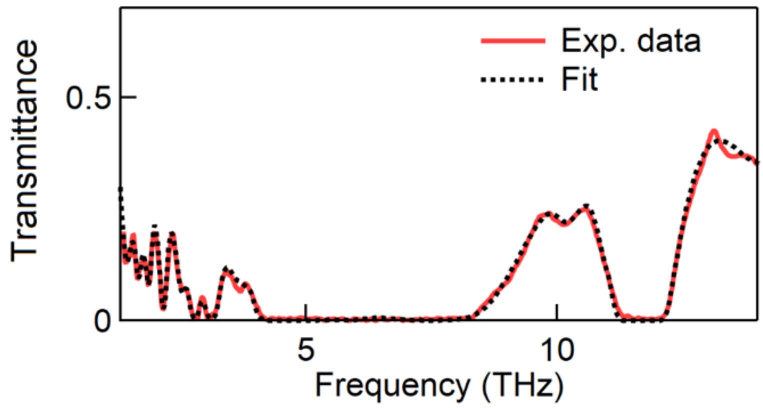

Supplementary Fig. 3. Transmission spectrum in the THz regime of a 30 µm-thick BBO crystal measured by FTIR spectroscopy. *Black dotted line denotes the fit curve.*

The real $\varepsilon_1(\omega)$ and imaginary part $\varepsilon_2(\omega)$ of complex dielectric function, depicted in Fig. 1d of the main manuscript, were found by fitting the optical spectra using the Kramers-Kronig (KK) consistent

dielectric function model with Reffit software[5]. The fit procedure for the transmission spectrum was based on the conventional dielectric function:

$$\varepsilon(\omega) = \varepsilon_\infty + \sum_i \frac{\omega_{p,i}^2}{\omega_{o,i}^2 - \omega^2 - i\Gamma_i\omega} \tag{S2}$$

Where $\omega_{0,i}$ (cm^{-1}), $\omega_{p,i}$ (cm^{-1}) and Γ_i (cm^{-1}) are the phonon strength (or plasma frequency), the eigenfrequency and the linewidth of the i-th Lorentz oscillator, respectively. ε_∞ is the high frequency dielectric constant. The black dashed line in Supplementary Fig. 3 is the fit and the corresponding parameters are listed in Supplementary Table 1. These parameters were used to model the BBO optical response in the finite-difference time-domain (FDTD) simulations (see Eq. 5 in Methods).

i	$\omega_{0,i}$ (cm^{-1})	$\omega_{p,i}$ (cm^{-1})	Γ_i (cm^{-1})	i	$\omega_{0,i}$ (cm^{-1})	$\omega_{p,i}$ (cm^{-1})	Γ_i (cm^{-1})
1	47.14	44.33	8.63	8	119.62	41.72	14.68
2	55.29	33.98	5.82	9	144.85	217.02	6.49
3	61.91	33.59	5.17	10	188.78	102.10	34.17
4	71.80	41.77	4.34	11	245.15	176.46	60.90
5	84.077	36.30	6.14	12	340.26	21.03	19.23
6	92.26	60.61	5.82	13	383.20	185.15	5.33
7	103.29	67.29	7.25	14	469.30	45.65	47.70
$\varepsilon_\infty = 2.89$							

Supplementary Table 1. Fit parameters of $\varepsilon(\omega)$ (Eq. S2). The parameters of the phonon at $\omega_{Q1} = 4.32\ THz$ are highlighted in blue.

Phonon resonances are found at $\omega_{Q1} = 4.32$ THz (144 cm^{-1}); $\omega_{Q2} = 5.65$ THz (189 cm^{-1}), $\omega_{Q3} =$ 7.34 THz (245 cm^{-1}) consistent with those in Ref.[4], which show peaks at $\omega_{Q1} = 4.65$ THz, $\omega_{Q2} =$ 5.65 THz, $\omega_{Q3} = 7.34$ THz. For ω_{Q1}, the discrepancy of 0.3 THz between the measurements here and those reported in Ref. [4] could originate from sample variation and/or instrument resolution. The current measurements use a 30 µm-thick uncoated BBO and the FTIR was performed with a resolution of 0.014 THz. While the measurements in Ref.[4] were performed using a BBO crystal with a 400 nm antireflection coating (which could also affect the THz transmission) and transmission spectra were collected with a lower frequency resolution (0.05 THz). The 30µm reference BBO in the current measurements was also evaluated using Raman spectroscopy, which yields E-symmetry modes at 143 cm^{-1}, 187 cm^{-1} and 245 cm^{-1}, which closely matches (to within 2 cm^{-1}) the values reported in Ref.[6].

Sample quality and orientation of the 300µm thick BBO used in the experiment were verified by Laue X-ray diffraction. Establishing the crystal orientation is essential for defining the crystallographic coordinate system (x', y', z') and its relation to the laboratory frame (x, y, z), which is required for both the DFPT calculations based on BBO crystal structure and the analytical model. The Laue pattern shows that the crystallographic a-axis corresponds to the y of the laboratory frame (ordinary axis), which coincides with the x'-axis in the crystal frame since $\phi = 90°$(Supplementary Fig. 4a). The crystal is *R3c* symmetric, so the *E* phonons are doubly degenerate. DFT calculations indicate that the THz-driven *E* mode at 144 cm^{-1} has two orthogonal in-plane dipole moments: E_1 along x' and E_2 along y' (Supplementary Fig. 4b). When the THz pump is polarised along the a-axis, the field couples only to the E_1 branch and the orthogonal E_2 branch is not excited.

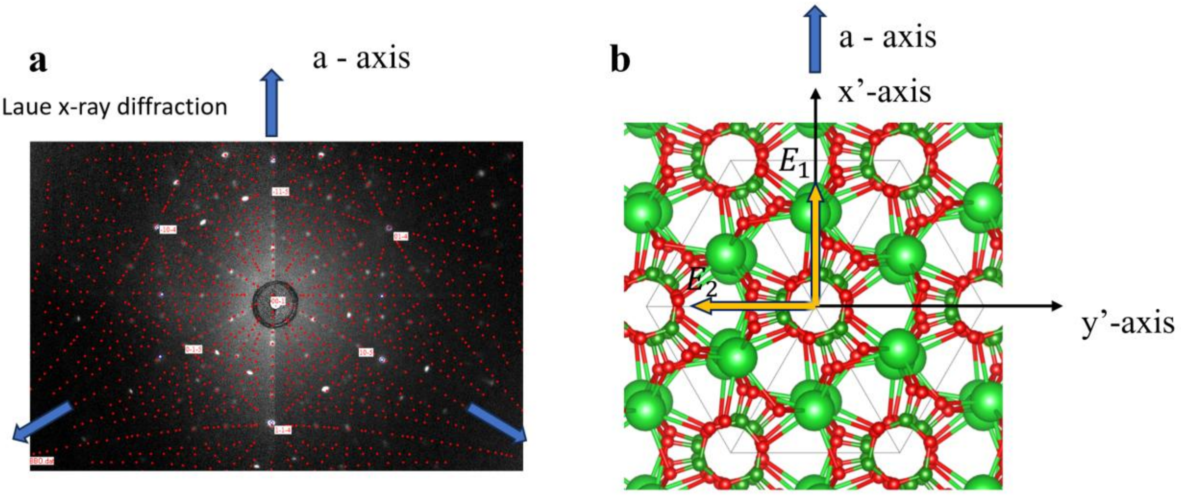

***Supplementary Fig. 4. Laue diffraction and sample orientation: a**, Laue diffraction pattern of the single crystal 300 μm thick BBO sample ($\theta = 29.3°$, $\phi = 90°$) used in the experiment to verify the experimental geometry. The THz electric field is aligned along the a-axis. In the Laue measurement, the crystal was rotated by $\sim -\theta°$ around the a-axis to confirm the $R3c$ symmetry. Red dots indicate simulated Laue diffraction peaks generated using Laue diffraction software. **b**, Crystal unit cell from DFPT calculations, shown in crystallografic coordinate system x', y' (in-plane) and z' (out-of-plane). The x'-axis corresponds to the crystallographic a-axis. The E-mode at 144 cm^{-1} is doubly degenerate, with branches E_1 and E_2 having dipole moment directions indicated by the orange arrows.*

4. Polarization angular dependence of static second harmonic generation

For BBO, the second-order nonlinear d_{NL} tensor has 3 independent coefficients[7] that are defined in the crystal coordinate system (x', y', z'):

$$d_{NL} = \begin{pmatrix} 0 & 0 & 0 & 0 & d_{15} & -d_{22} \\ -d_{22} & d_{22} & 0 & d_{15} & 0 & 0 \\ d_{15} & d_{15} & d_{33} & 0 & 0 & 0 \end{pmatrix}, \tag{S3}$$

where $d_{15} = 0.16$ pm/V, $d_{22} = -2.2$ pm/V, $d_{33} \sim 0$.

In the laboratory coordinate system, the FF is linearly polarized and propagates in the z-direction. The polarization is defined by the angle α of the polarization vector with respect to the vertical, y-axis, which corresponds to the ordinary axis of the static BBO crystal in the laboratory frame. In this case:

$$E_{lab}^{FF}(\alpha) = E_0\, u, \tag{S4}$$

where $u = (\sin\alpha, \cos\alpha, 0)$ is the polarization vector and E_0 is the amplitude of the FF beam. Experimentally, the polarisation is varied using a half-wave plate.

The polarization vector in the crystal frame is found using the transformation $u_{xtal} = Ru$ where θ is the polar angle - which is the angle of the lab frame z-axis with respect to the crystal c-axis - and ϕ is the azimuthal angle -which is the angle of the lab frame x- and y-axes with respect to the crystal ordinary axis (for the BBO crystal used in the experiments, $\theta = 29.3°$ and $\phi = 90°$).

$$R = \begin{pmatrix} -\cos\theta\cos\phi & \sin\phi & \cos\phi\sin\theta \\ -\cos\theta\sin\phi & -\cos\phi & \sin\theta\sin\phi \\ \sin\theta & 0 & \cos\theta \end{pmatrix} \tag{S5}$$

With this transformation, it follows that the effective nonlinear tensor d_{eff} in the laboratory frame, is:

$$d_{eff} = R^T d_{NL}\, f_{3\to6}(Ru), \tag{S6}$$

Where $f_{3\to6}$ is the operator[8] that maps the square of the electric field components (x', y', z') onto $(x'^2, y'^2, z'^2, 2y'z', 2x'z', 2x'y')$ in the crystal frame.

For Type-I phase matching $(o + o \to e)$, the SH harmonic is polarized along the extraordinary axis (x-axis in the lab frame). Therefore, for this component one finds:

$$E_{SH}^{x}(\alpha) \propto (\sin\theta\, d_{15} - \cos\theta \sin 3\phi\, d_{22}) \cos^2\alpha\, E_0^2 \tag{S7}$$

For this particular SH component, the nonlinear tensor elements in d can be written as a scalar coefficient d_{eff}.

$$E_{SH}^{x}(\alpha) \propto d_{eff} \cos^2\alpha\, E_0^2 \tag{S8}$$

The polarisation-dependent Type-I generated SH intensity $I_{SH,0}$ is then proportional to d_{eff}^2 and $\cos^4\alpha$ as experimentally observed (Fig. 3b in the main manuscript).

5. Analytical model: THz Vibrational Modulation of Optical Properties

The THz-driven phonon modulates the refractive index ellipsoid η_i through the electro-optic tensor r_{ij}. The BBO crystal structure is *3m,* therefore the electro-optic tensor in the Voigt notation in the crystal coordinate system (x', y', z') is written as[8]:

$$r_{ij}^{EO} = \begin{pmatrix} 0 & -r_{22} & r_{13} \\ 0 & r_{22} & r_{13} \\ 0 & 0 & r_{33} \\ 0 & r_{51} & 0 \\ r_{51} & 0 & 0 \\ -r_{22} & 0 & 0 \end{pmatrix}. \tag{S9}$$

With an applied field, the components of the index of refraction ellipsoid undergo a variation $\Delta\eta_i = r_{ij}^{EO} \cdot E_j$, where E_j are the electric field components in the crystal frame driving the electro-optic effect. The electro-optic effect generated by a temporally modulated electric field can occur through two distinct mechanisms: electronic and vibrational that are described by "electronic" electro-optic coefficient r_{ij}^{el} and the "vibrational" electro-optic coefficient r_{ij}^{vib}, Ref. [9]. When a THz electric field E_{THz} is applied, the change in the index of refraction ellipsoid tensor elements can then be rewritten as: $\Delta\eta_i = \left(r_{ij}^{el} + r_{ij}^{vib}\right) \cdot E_{THz,j}$. In BBO, the vibrational contribution is known to be dominant – even for off-resonant electric fields at low frequencies and it is strongly enhanced when approaching the phonon frequency at ~ 4.3 THz.[9] Evidence of this effect is found in the experiment through the observation of a significant increase in SH modulation on resonance, when the THz pump spectral components overlap with the phonon frequency (2a-b of the main manuscript). Therefore, near the phonon resonance, the vibrational contribution can be considered to be dominant and only $r_{ij}^{EO} = r_{ij}^{vib}$ is considered in the electro-optic tensor[9]. Since the phonon amplitude scales as $Q_j \propto Z E_{THz,j}$, where Z is the phonon effective charge, the vibrational term $r_{ij}^{vib} E_{THz,j}$ can be rewritten as $r_{ij} Q_j$. Here, r_{ij} is the phonon–optic

coupling coefficient, which describes the modulation of the refractive-index ellipsoid element as a function of the phonon amplitude.

In the laboratory frame (x, y, z), the extraordinary axis is along x and the ordinary axis is along y, while the light propagates along the z direction. In the experiment, the THz electric field polarisation is fixed and aligned along the ordinary, y-axis. The THz polarization in the Jones formalism is $E_{THz} = (0,\ E_{THz,y},\ 0)$. The transformation matrix R is used to convert the THz components from the laboratory frame (x, y, z) to the crystallographic frame (x', y', z'):

$$R = \begin{pmatrix} -\cos\theta\cos\phi & \sin\phi & \cos\phi\sin\theta \\ -\cos\theta\sin\phi & -\cos\phi & \sin\theta\sin\phi \\ \sin\theta & 0 & \cos\theta \end{pmatrix} \tag{S10}$$

The THz electric field in the crystal frame is then $E_{THz}^{xtal} = R\ E_{THz}$. Under coherent phonon excitation, the index ellipsoid becomes $\eta_Q = \eta_0 + \Delta\eta_Q$, where η_0 is the index ellipsoid at the equilibrium [7]. In crystal frame (x', y', z'), η_0 can then be written in matrix form as:

$$\eta_0 = \begin{pmatrix} \frac{1}{n_{x'}^2} & 0 & 0 \\ 0 & \frac{1}{n_{x'}^2} & 0 \\ 0 & 0 & \frac{1}{n_{z'}^2} \end{pmatrix}, (n_{x'} = n_{y'}). \tag{S11}$$

Given that BBO is uniaxial, for the modulation of the index ellipsoid $\Delta\eta_Q$, only the phonon-optic coupling coefficient r_{22}, which is related to r_{ij}^{EO}, is significant and the others can be neglected[8,10].

As the phonon dipole moment is along the THz electric field direction[3], the phonon amplitude is $Q_0 \propto Z\ E_{THz}^{xtal} \equiv Z E_{THz,y'}$. Thus, the modulated refractive index ellipsoid, calculated from the electro-optic tensor in Eq. S9, can be written in the matrix form as:

$$\eta_Q = \begin{pmatrix} \frac{1}{n_{x'}^2} + r_{22}Q_0\cos\phi & -r_{22}Q_0\sin\phi & 0 \\ -r_{22}Q_0\sin\phi & \frac{1}{n_{x'}^2} - r_{22}Q_0\cos\phi & 0 \\ 0 & 0 & \frac{1}{n_{z'}^2} \end{pmatrix}. \tag{S12}$$

Since $\phi = 90°$, a finite value of phonon amplitude Q_0 leads to the emergence of off-diagonal terms, and the crystal becomes biaxial associated with an optical anisotropy in the x'-y' plane, as the degeneracy between x' and y' directions is lifted.

The index ellipsoid in the crystal frame can be written as $\eta_Q = \eta_0 - r_{22}Q_0 \cdot A$, where:

$$A = \begin{pmatrix} 0 & 1 & 0 \\ 1 & 0 & 0 \\ 0 & 0 & 0 \end{pmatrix}. \tag{S13}$$

The dynamical refractive index ellipsoid in the laboratory frame η_{THz}^{Lab} can then be determined upon cartesian axis transformation: $\eta_{THz}^{Lab} = R^T\eta_0 R - r_{22}Q_0\ R^T A R = R^T\eta_0 R - \epsilon\ R^T A R$, with $\epsilon = r_{22}Q_0$ and R^T the transpose of R. The two terms of η_{THz}^{Lab} are analysed separately:

$$R^T \eta_0 R = \begin{pmatrix} \frac{\cos^2\theta}{n_{x'}^2} + \frac{\sin^2\theta}{n_{z'}^2} & 0 & \cos\theta\sin\theta\left(\frac{1}{n_{z'}^2} - \frac{1}{n_{x'}^2}\right) \\ 0 & \frac{1}{n_{x'}^2} & 0 \\ \cos\theta\sin\theta\left(\frac{1}{n_{z'}^2} - \frac{1}{n_{x'}^2}\right) & 0 & \frac{\sin^2\theta}{n_{x'}^2} + \frac{\cos^2\theta}{n_{z'}^2} \end{pmatrix}, \tag{S14}$$

and

$$\epsilon\, R^T A R = \epsilon \begin{pmatrix} 0 & -\cos\theta & 0 \\ -\cos\theta & 0 & \sin\theta \\ 0 & \sin\theta & 0 \end{pmatrix}. \tag{S15}$$

With the definition $\frac{1}{n_e(\theta)^2} = \frac{\cos^2\theta}{n_{x'}^2} + \frac{\sin^2\theta}{n_{z'}^2}$ and $\frac{1}{n_o^2} = \frac{1}{n_{x'}^2}$, and reducing the analysis to two dimensions, considering that the light polarization lies in the x-y plane of the laboratory frame, it follows that:

$$\eta_{THz}^{Lab} = \begin{pmatrix} \frac{1}{n_e(\theta)^2} & \epsilon\cos\theta \\ \epsilon\cos\theta & \frac{1}{n_o^2} \end{pmatrix} \tag{S16}$$

This dynamical index ellipsoid in the lab frame is diagonalized by a rotation of γ_0, and the new refractive indexes are:

$$n_o^{THz} = n_o + (r_{22}Q_0)^2 n_o^3 \gamma_0/2 \tag{S17}$$

and

$$n_e^{THz} = n_e(\theta) - (r_{22}Q_0)^2 n_e^3(\theta)\,\gamma_0/2, \tag{S18}$$

with $\gamma_0 = \kappa\,\epsilon + O[\epsilon]^2 = \kappa\, r_{22}Q_0$ with $\kappa = \frac{\cos\theta}{\frac{1}{n_e(\theta)^2} - \frac{1}{n_o^2}}$.

The primary result is that the dynamical principal axes of the crystal in the laboratory frame are no longer aligned with the x and y crystal axes upon phonon excitation with amplitude Q_0. Instead, they are rotated by an angle γ_0, which is proportional to Q_0. Importantly, the magnitude of the refractive indices are not significantly altered by Q_0, since the correction is to 3rd-order $(r_{22}Q_0)^2\gamma_0 \propto (r_{22}Q_0)^3$.

6. Analytical model: Vibrational Modulation of Second Harmonic Intensity

For the Type-I phase-matched BBO crystal ($\phi = 90°, \theta = 29.3°$), the SHG intensity is proportional to $\left|E_y^{FF}\right|^4$, where E_y^{FF} is the projection of the amplitude of the 800 nm FF on the lab-frame y-axis, which is parallel to the unperturbed crystal-frame ordinary axis. The SH will be polarised along the crystal extraordinary axis, which is parallel to the lab-frame x-axis.

A linearly polarised FF propagating along the z-direction in the laboratory frame has field components (x, y) given by the following expression:

$$E^{FF}(\alpha) = E_0^{FF}\,(\sin\alpha, \cos\alpha) \tag{S19}$$

where α is the angle between that y-axis and the input polarisation.

Due to the birefringence of the BBO crystal, for input fundamental fields that are not aligned with the principal axes, the polarisation will evolve with propagation distance ζ in the crystal. In the static case, at equilibrium, after travelling a distance ζ, the evolution of the field components is determined using the linear phase retardation matrix $\Gamma(\zeta)$ such that:

$$E^{FF}(\zeta,\alpha) = \Gamma(\zeta)E^{FF}(\alpha) \quad \text{where} \quad \Gamma(\zeta) = \begin{pmatrix} e^{i2\pi n_e\zeta/\lambda} & 0 \\ 0 & e^{i2\pi n_o\zeta/\lambda} \end{pmatrix} \tag{S20}$$

In this case, and in the undepleted pump approximation, the Type-I $(o + o \rightarrow e)$ SH intensity from a crystal length L as a function of input polarisation α can be written as:

$$I_{SH,0}(L,\alpha) \propto \left| \int_0^L \left(E_y^{FF}(\zeta,\alpha)\right)^2 e^{-ik_{n_{e,2\omega}}\zeta} d\zeta \right|^2 \tag{S21}$$

And upon substitution:

$$I_{SH,0}(L,\alpha) \propto \left| \int_0^L (E_0^{FF}\cos\alpha)^2 e^{i4\pi n_{o,\omega}\zeta/\lambda} e^{-ik_{n_{e,2\omega}}\zeta} d\zeta \right|^2 \tag{S22}$$

For perfect Type-I phase matching, $\Delta k = 0, n_{o,\omega} = n_{e,2\omega}$ and the exponential term reduces to 1, and the SH intensity is:

$$I_{SH,0}(L,\alpha) \propto L^2(E_0^{FF})^4\cos^4\alpha \tag{S23}$$

In the dynamic case, the principal axes of the BBO crystal are rotated, however, the $\chi^{(2)}$ tensor remains unchanged. This is confirmed by DFPT calculations, which show that even for relatively large phonon amplitudes the variation of the $\chi^{(2)}$ tensor elements remains negligible with respect to the static d_{22} value (see Supplementary Fig. 6). Therefore, the relevant components of the input field remain the x and y-components in the laboratory frame.

Again, the linearly polarized input field can be expressed as:

$$E^{FF} = E_0^{FF}(\sin\alpha, \cos\alpha) \tag{S24}$$

However, now, upon THz excitation the principal axes are rotated by an amount γ. To calculate the propagation phase-advance, the components are rotated onto the new principal axes coordinate system by R_γ, before applying the linear phase retardation and then rotated back to the original lab-frame coordinate system to find the FF components as a function of the propagation distance ζ. In this case the fundamental field components are given by the expression:

$$E^{FF}(\zeta,\gamma) = R_\gamma^T\Gamma(\zeta)R_\gamma E^{FF} \tag{S25}$$

Here, R_γ is the rotation matrix associated with the rotation of the ordinary and extraordinary axes (see Fig. 4a of the main text), and is written as:

$$R_\gamma = \begin{pmatrix} \cos\gamma & -\sin\gamma \\ \sin\gamma & \cos\gamma \end{pmatrix}, \tag{S26}$$

and R_γ^T is its transpose.

If the rotation of the principal axes is considered to be constant (γ_0) and again in the undepleted pump approximation, the intensity of the SH polarized along lab-frame x-axis after propagating to a depth z inside the crystal is:

$$I_{SH,THz}(z,\alpha,\gamma_0) \propto \left| \int_0^z \left(E_y^{FF}(\zeta,\gamma_0) \right)^2 e^{-ik_{n_{e,2\omega}}\zeta} d\zeta \right|^2. \qquad \text{(S27)}$$

Upon substitution, three terms appear in the integral with distinct arguments in the exponential corresponding to Type-0, Type-I and Type-II wave mixing processes. If the Type-0 and Type-II processes are neglected due to a lack of phase matching, i.e. $n_{e,\omega} \neq n_{e,2\omega}$ and $n_{e,\omega} + n_{o,\omega} \neq 2n_{e,2\omega}$, and only the Type-I process is considered, the integral in the intensity term becomes:

$$E_{SH,THz}(z,\alpha,\gamma_0) \propto E_0^2 \cos^2(\alpha - \gamma_0) \frac{2\sin(\Delta k z/2)}{\Delta k} e^{i\Delta k z} \qquad \text{(S28)}$$

Where $\Delta k = (\omega/c)\left(n_{0,\omega} - n_{e,2\omega}\right) = (\omega/c)\Delta n$.

For small rotations of the principal axes ($\gamma_0 \ll \pi/2$), the expression for $E_{SH,THz}$ can be expanded.

$$E_{SH,THz}(z,\alpha,\gamma_0) \propto E_0^2 z \,\mathrm{sinc}(\Delta k z/2)\, e^{i\Delta k z} \cos\alpha \,[\cos\alpha + 2\gamma_0 \sin\alpha + O[\gamma_0^2]] \qquad \text{(S29)}$$

And, finally, the SH intensity to 1st-order in γ_0 becomes:

$$\begin{aligned} I_{SH,THz}(z,\alpha,\gamma_0) &= \left|E_{SH,THz}(z,\alpha,\gamma_0)\right|^2 \\ &\propto E_0^4 z^2 \,\mathrm{sinc}^2(\Delta k z/2)\,[\cos^4\alpha + 4\gamma_0 \cos^3\alpha \sin\alpha] \end{aligned} \qquad \text{(S30)}$$

Assuming perfect Type-I phase matching, $\Delta k = 0$, the intensity of the SH is given by the expression:

$$I_{SH,THz}(z,\alpha,\gamma_0) \propto E_0^4 z^2 \cos^3\alpha \,(\cos\alpha + 4\,\gamma_0 \sin\alpha) \qquad \text{(S31)}$$

At equilibrium, $\gamma_0 = 0$, and the conventional expression for SH intensity is recovered, in agreement with the angular dependence observed in the experiment (Fig. 3b of the main manuscript).

Based on equation S31, the SH intensity modulation can be approximated as:

$$\Delta I_{SH.THz} = I_{SH,THz} - I_{SH,0} \approx 4\rho E_0^4 z^2 \gamma_0 \cos^3\alpha \sin\alpha \qquad \text{(S32)}$$

where $\rho = \frac{\omega^2 d_{NL}^2}{n_\omega^2 c^2}$ is the proportionality constant and, d_{22} the nonlinear coefficient of Type-I SHG[11].

In the dynamical case, for a finite phonon amplitude Q_0 and rotation of the principal axes γ_0, the analytical expression for the SH intensity modulation in Eq. S32 matches the angular dependence observed in the experiment.

In the presence of optical absorption due to IR-active vibrations, the THz electric field undergoes exponential attenuation within the crystal. As a results, the phonon amplitude decreases as a function of the propagation depth ζ and can be approximated with an exponential decay, $Q(\zeta) = Q_0\, e^{-\zeta/\delta_{ph}}$, where δ_{ph} is the phonon propagation depth. Consequently, along the propagation, the parameter γ, being proportional to Q, also undergoes exponential depletion according to $\gamma(\zeta) = \gamma_0 e^{-\zeta/\delta_{ph}}$. Then, including the propagation dependence of $\gamma(\zeta)$, the SH field $E_{SH,THz}$ can be written as:

$$E_{SH,THz}(z,\alpha,\gamma_0,\delta_{ph}) = \int_0^z \left(E_y^F(\zeta,\gamma_0,\delta_{ph}) \right)^2 e^{-\frac{i2n_o\pi\zeta}{\lambda/2}} d\zeta \qquad \text{(S33)}$$

In this case, the expression for the SH intensity modulation for $z = L$ is found to be:

$$\Delta I_{SH,THz} = 4\rho E_0^4\, L\, \gamma_0 is\ \cos^3\alpha \sin\alpha\, \Lambda(\delta_{ph}, L). \tag{S34}$$

After substituting the static SH intensity at $\alpha = 0$, $I_{SH,0}(0) = \rho E_0^4 z L^2$, the modulation becomes:

$$\Delta I_{SH,THz}(\alpha) = 4\frac{I_{SH,0}(0)}{L}\, \gamma_0\ \cos^3\alpha \sin\alpha\, \Lambda(\delta_{ph}, L), \tag{S35}$$

which corresponds to Eq. 3 of the main manuscript.

Equation S35 is similar to Eq. S32, but it includes the depletion factor:

$$\Lambda(\delta_{ph}, z = L) = \left(4e^{-\frac{z}{\delta_{ph}}}\right) \cdot \left(\frac{-4\pi^2\Delta n^2 + 4e^{\frac{z}{\delta_{ph}}}\pi^2\Delta n^2 - \frac{\lambda^2}{\delta_{ph}{}^2} + \frac{\lambda^2}{\delta_{ph}{}^2 \cos\left(\frac{2\pi z\Delta n}{\lambda}\right)} - \frac{2\pi\Delta n\lambda}{\delta_{ph} \sin\left(\frac{2\pi z\Delta n}{\lambda}\right)}}{\frac{4\pi^2\Delta n^2}{\delta_{ph}} + \frac{\lambda^2}{\delta_{ph}{}^3}} \right) \tag{S36}$$

To evaluate the depletion factor, the wavelength-dependent refractive indices for BBO at room temperature were taken from Ref.[12].

Eq. S35 shows that the SHG modulation intensity $\Delta I_{SH,THz}(\alpha)$ scales with the parameter γ_0, and therefore with the phonon amplitude Q_0, as expected and reproduces the same angular dependence observed experimentally. This equation has been used to fit the polarization-resolved differential SHG modulation (see Fig. 4b in the main text) and to extract the value of γ_0. A key quantity in this analysis is the phonon penetration depth, δ_{ph}, which is determined to be 30.2 μm by finite-difference time-domain (FDTD) simulations (Fig. 4c in the main text).

7. Higher-order Nonlinear Processes

Alongside the phonon-mediated electro-optic modulation of the optical axis - a $\chi^{(2)}$ process that modulates the SHG through phase matching – higher-order THz-mediated $\chi^{(3)}$ effects. These $\chi^{(3)}$ processes have been described within the framework of Terahertz-Field–Induced Second Harmonic (TFISH). It should be noted, however, that in previous TFISH works[13–16], either the phonons were not resonantly excited, or critically, the SHG process was phase-mismatched. In this case, the frequency conversion is confined to a coherence length that is typically on the order of microns – commensurate with a few wavelengths of the fundamental beam. Within this short-interaction regime the THz-driven phonons directly modulate the second-order nonlinearity resulting in a $\chi^{(3)}$ effect, while second-order electro-optic variation of the refractive index ellipsoid and its impact on non-existent phase matching conditions are negligible.

In the current experiment, $\chi^{(3)}$ effects are found to have negligible impact on SHG modulation under optimal phase matching conditions, as they cannot reproduce the angular dependence observed in the experiment, the $\chi^{(3)}$ SHG modulation increases and decreases uniformly as a function of time delay for all input polarisations (rather than exhibiting a vice-versa behaviour between neighbouring lobes as observed in the experiment), and finally, the magnitude of the $\chi^{(3)}$-effects on the SHG intensity modulation are much smaller to those observed in the experiment.

To isolate and study the $\chi^{(3)}$-effects, additional experiments were performed in a c-cut (θ = 0°) BBO crystal (MolTech GmbH, 500 µm thick), which is far from Type-I phase-matching conditions (Supplementary Fig. 5a-b). The crystal quality and orientation were verified by Laue x-ray diffraction.

In this configuration, the THz and the NIR pulses propagate along the optic axis (c-axis) and the refractive index is n_o along x- and y-axes in the laboratory frame. Therefore, the crystal is isotropic and the coherence length is identical for all input polarization states.

In the phase-mismatched case, the equilibrium SHG conversion is two orders of magnitude lower than in the phase-matched case and the nonlinear coefficient d_{22} dominates the SHG conversion. Analytically, the SH intensity along the lab-frame x-axis is, $I_{SHG,x} \propto |d_{22}E_x^2 - d_{22}E_y^2|^2$, where $E_x = E_0^{FF} \sin\alpha$ and $E_y = E_0^{FF} \cos\alpha$ are the projections of fundamental field E^{FF} along the lab-frame x- and y-axes. From the expression, a 4-fold symmetry in input polarisation angle α is expected, with local maxima on the x- and y-axes. Indeed, at equilibrium in the phase-mismatched case, the observed SHG has a polarization dependence in agreement with the analytical expression (Fig S5c).

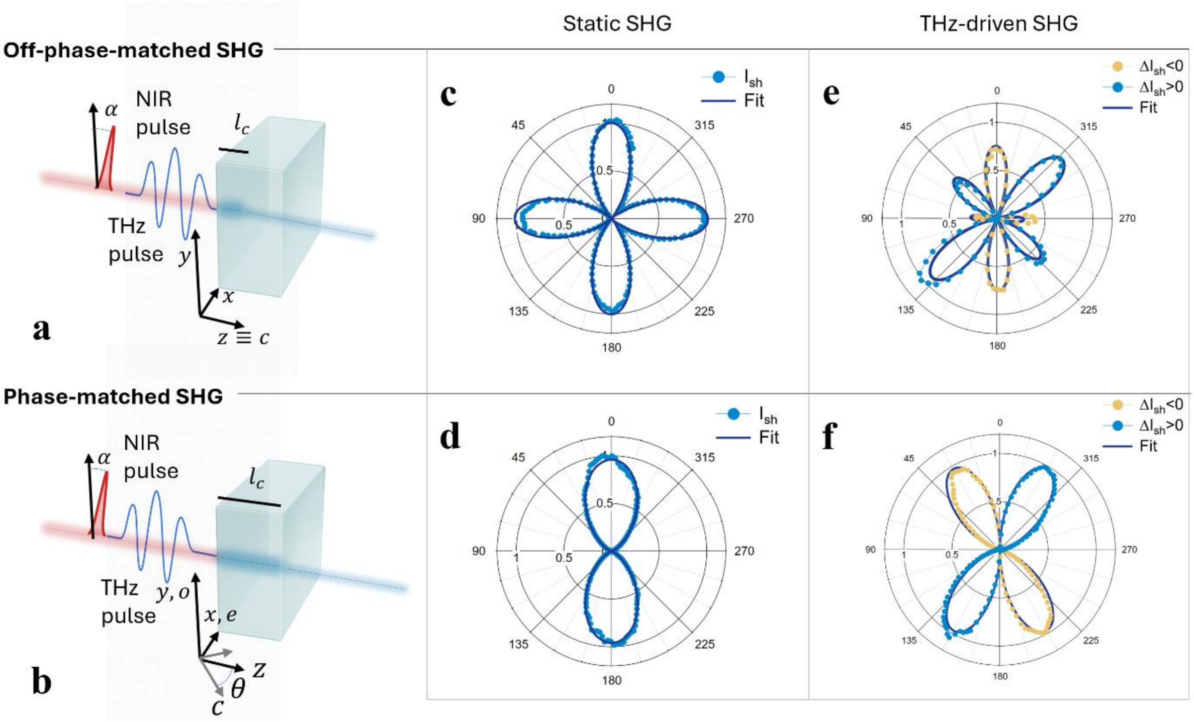

***Supplementary Fig. 5. Isolating the TFISH contribution to THz-driven SHG modulation. a**, Off–phase-matched geometry: the NIR and THz pulses propagate along the crystal c axis; SHG is collected along y. **b**, Phase -matched SHG geometry. **c**, Static "off-phase-matched" SHG: experimental data and fit curve with the function: $I_{SHG} \propto |d_{22}E_x^2 - d_{22}E_y^2|^2$. **d**, Static phase-matched SHG: experimental data with fit: $I_{SHG} \propto |d_{22}E_y^2|^2$, where the E_x^2 is not present because not phase-matched. **e**, THz-induced SHG modulation in the off–phase-matched case: data and fit: $\Delta I_{SHG} \propto a_1E_x^4 + a_2E_y^4 + a_3E_x^2E_y^2 + a_4E_x^3E_y + a_5E_xE_y^3$, modelling TFISH-driven modulation of the $\chi^{(2)}$ tensor components, see Ref.[14]. The a_3 is dominant and produces lobes oriented at 45°; a_4 and a_5 are over one order of magnitude smaller. The maximum SHG intensity modulation is below 1% at the maximum THz field. **f**, THz-induced SHG modulation under phase-matched conditions: the angular symmetry differs qualitatively from panel **e** and cannot be described by TFISH alone.*

In contrast, when equilibrium SHG measurements are performed under optimal Type-I phase matching conditions, only the SH due to the Type-I process (SH along the extraordinary in the lab frame, x-axis) is generated by the component of the FF field projected along the ordinary, y-axis $(o + o \rightarrow e)$. Contributions from the other input fundamental field components are negligible due to a lack of phase matching. Analytically, this results in a static SHG signal $I_{SHG} \propto |d_{22}E_x^2|^2 \propto \cos^4\alpha$ with 2-fold angular symmetry (see Supplementary Fig. 5d).

In the dynamical case, the THz excitation modulates the intensity of the SH. The effect can be considered from two perspectives, either the THz field can be viewed to modulate the 2nd-order

nonlinear susceptibility such that $\chi^{(2)} \rightarrow \chi^{(2)}(t)$, or the effect can be seen to occur through a 4-wave mixing process involving the 3rd-order nonlinear susceptibility $\chi_0^{(3)}$ and the THz and fundamental fields. The second scenario is commonly used in the description of TFISH experiments and is why THz-Field Induced Second Harmonic is often known as a 3rd-order χ^3-effect. Within the TFISH framework, the intensity of the SHG signal has three terms, a time-dependent heterodyne term which depends linearly on the THz electric field, a homodyne term and a DC offset term[17]:

$$I_{SHG}(t) \propto \left|\left(\chi^{(2)} + \chi^{(3)} E_{THz}(t)\right) E_{FF}^2\right|^2$$
$$\propto \underbrace{\left|\chi^{(2)} E_{FF}^2\right|^2}_{DC} + \underbrace{2Re\left(\chi^{(2)}\chi^{(3)} E_{THz}(t) E_{FF}^4\right)}_{Heterodyne \propto E_{THz}(t)} + \underbrace{\left|\chi^{(3)} E_{THz}(t) E_{FF}^2\right|^2}_{Homodyne \propto |E_{THz}(t)|^2} . \tag{S37}$$

In the alternative perspective, where the THz electric field is assumed to modulate the 2nd-order susceptibility tensor, $\chi^{(2)}(t)$ can be written as:

$$\chi^{(2)}(t) = \chi^{(2)} + \Delta\chi^{(2)}(t) \text{ and } I_{SHG}(t) \propto \left|\chi^{(2)}(t) E_{FF}^2\right|^2, \tag{S38}$$

where $\chi^{(2)}$ is the static nonlinear susceptibility tensor and $\Delta\chi$ is the THz induced, time-dependent modulation.

The pictures are equivalent, as the time-dependent change in the nonlinear susceptibility is assumed proportional to the THz field, $\Delta\chi^{(2)}(t) \sim \chi^{(3)} E_{THz}(t) \sim \chi^{(3)} Q(t)$.

If only a single input FF polarization is used, the heterodyne term in the time-dependent intensity might erroneously be considered as the source of the SH modulation in the phase-matched experiment. The effect is linearly dependent in strength on the THz electric field, it is phase-locked to the THz electric field, and it has a frequency dependence that matches the THz-driven phonon. However, the strength of the 3rd-order TFISH effect is expected to be much weaker than the 2nd-order effect, and perhaps even more conclusively, when the angular symmetry of the intensity modulation is included in the analysis, it can be shown that 3rd-order processes cannot be the underlying source of the observed dynamics in the phase-matched case.

To investigate the polarization dependence, the perspective employing a time-dependent $\chi^2(t)$ will be used for analysis and interpretation of the dynamical SH signal in both the phase-matched and phase-mismatched geometries. By convention, the nonlinear susceptibility is expressed in terms of the nonlinear coefficient $d = \frac{1}{2}\chi^{(2)}$, so that the dynamical SH field amplitude is:

$$E_{SH,THz}^{xtal} \propto (d_0 + \Delta d) E_{xtal}^{FF} E_{xtal}^{FF}, \tag{S39}$$

where E_{xtal}^{FF} is the fundamental field in the crystal-frame, d_0 is the nonlinear coefficient at the equilibrium and Δd is the modulation induced by the THz field.

By applying a coordinate transformation from the lab-frame to crystal-frame, evaluating the SH fields that are generated in the BBO and then transforming back to the lab-frame, the resultant SH field components can be related to the input polarisation of the fundamental field in the lab frame, which is defined by α,

$$E_{SH,THz}^{lab}(\alpha) = R^T (d_0 + \Delta d) f_{3\rightarrow 6}\left(R\left(E_{FF}^{lab}(\alpha)\right)\right). \tag{S40}$$

Here, $f_{3\rightarrow 6}$ is the operator that maps vector components (x', y', z') of the input field onto the $(x'^2, y'^2, z'^2, 2y'z', 2x'z', 2x'y')$ terms corresponding to the square of the input field. The components of the fundamental field in the lab frame are, $E_0^{FF}(\sin\alpha, \cos\alpha, 0)$, with α the polarization angle with respect to the y-axis. R is the rotation matrix that maps the field components from the lab frame to the crystal frame and is a function of the crystal geometry $(\theta = 29.3, \phi = 90)$.

Finally, the SH intensity as a function of input polarisation can be written as:

$$I_{SH,THz}(\alpha) \propto \left|E_{SH,THz}^{lab}(\alpha)\right|^2 \propto \left|R^T(d_0 + \Delta d) f_{3\to 6}\left(R\left(E_{FF}^{lab}(\alpha)\right)\right)\right|^2 \tag{S41}$$

The output intensity in the dynamical case contains interference terms involving the static 2nd-order nonlinearity and time-dependent 2nd-order nonlinearity – or equivalently the $\chi^{(3)}$ nonlinear susceptibility.

When the x-component in the lab frame is isolated with a polariser (corresponding to the extraordinary axis in the phase matched geometry), and for generality if all elements in the Δd tensor are allowed to be non-zero, the polarisation dependent intensity difference includes the five following terms ($E_z = 0$):

$$\begin{aligned}\Delta I_{SH,THz}^{x}(\alpha) &= I_{SH,THz}^{x} - I_{SH,0}^{x} \\ &\propto (a_1)E_x^4 + (a_2)E_y^4 + (a_3)E_x^2E_y^2 + (a_4)E_x^3E_y + (a_5)E_xE_y^3,\end{aligned} \tag{S42}$$

where:

$$E_x = E_0^{FF}\sin\alpha \quad ; \quad E_y = E_0^{FF}\cos\alpha. \tag{S43}$$

In Eq. S42, the $a_1,..,a_5$ coefficient depends on the static and modulated nonlinear tensor elements. Given the initial symmetry of the BBO crystal ($d_{15}, d_{22}, d_{33} \neq 0$), the THz induced variation in the intensity of the second harmonic can have an angular dependence with terms proportional to, $\cos^4\alpha, \sin^4\alpha, \cos^2\alpha\sin^2\alpha, \cos\alpha\sin^3\alpha$ and $\cos^3\alpha\sin\alpha$, and the proportionality will depend to 1st or 2nd order on the time-dependent second order nonlinearity, or the 3rd- order susceptibility $\chi^{(3)}$.

The angular dependence of the SH intensity modulation in the phase-mismatched case is shown in Supplementary Fig. 5e. Here, as previously stated, the TFISH, $\chi^{(3)}$-effects are isolated and the angular dependence can be fit with an equation of the form in S42. The dominant fit parameters are found to be associated with the $\cos^4\alpha, \sin^4\alpha$ terms, which scale the lobes on the x- and y-axes, and the $\cos^2\alpha\sin^2\alpha$ term, which scales the lobes along the diagonals. Small adjustments to the rotation of the polar fit can be made using the remaining terms. In the time-domain, the onset of THz excitation results in a uniform decrease of the SHG signal on the axes, $\Delta \mathrm{I}_{\mathrm{SHG}} < 0$, which is consistent with a THz-induced decrease of the d_{22} coefficient. At the same time, the onset of THz excitation results in a uniform increase of the SHG signal on the diagonals, $\Delta I_{SHG} > 0$.

The angular dependence in the Type-I phase-matched case is shown in Supplementary Fig. 5f. Here, in contrast to the phase-mismatched case, the strongest variation in the SH intensity is found at $\pm 30°$ and the signal on the axes is minimised. Moreover, the sign of the effect alternates between lobes such that if one lobe experiences an increase in SH intensity, the neighbouring lobe exhibits a decrease. If the dynamics were explained by TFISH or, equivalently, $\chi^{(3)}$ effects, one would expect that the $\cos^4\alpha, \sin^4\alpha$ and $\cos^2\alpha\sin^2\alpha$ terms would all play a role in the SH modulation, yet there is no clear signature of any of these terms in the angular dependence of the phase-matched measurements. This is especially notable for the $\sin^4\alpha$ term, which involves a heterodyne term that mixes two Type-I phase matched processes, and would be expected to have the strongest response.

Instead, only the $\cos^3\alpha\sin\alpha$ term from the TFISH scenario is present in the phase-matched angular dependence. A closer look at the elements in the proportionality coefficient of the $\cos^3\alpha\sin\alpha$ term, reveals component that is linear in THz field strength and scales with $O(\Delta d)$ or $O\left(\chi_0^{(3)}\right)$, and a component that is quadratic in THz field strength and scales with $O^2(\Delta d)$ or $O^2\left(\chi_0^{(3)}\right)$.

The homodyne contribution can be excluded because the SH intensity modulation varies linearly with the THz field strength (Fig. 2c of the main manuscript). Furthermore, as the homodyne contribution is proportional to $\sim Q^2$, it would need to be driven by a phonon at half the observed modulation frequency. However, in the experiment, when the driving THz was filtered to excite only a phonon at 2.15THz, the intensity modulation is nearly extinguished (Fig. 2, main manuscript).

There are also reasons to suggest that the heterodyne term does not contribute significantly to the phase-matched intensity modulation either. Importantly, the heterodyne interference term involves an SH component proportional to d that results from the Type-I phase-matched process, but the other component, proportional to the Δd time-dependent nonlinearity, results from a Type-II process, which is NOT phase matched.

In summary, the angular dependence in the phase-matched case does not show the features one would expect from the phase-mismatched reference measurements, and the features that are expressed do not appear to have originated from a $\chi^{(3)}$ process.

The features in the phase-matched case are however fit perfectly by the cascaded 2nd-order model involving electro-optic modification of the index of refraction ellipsoid and its effect on phase-matching in the usual SHG process with static 2nd-order nonlinearity.

8. Numerical estimate of the $\chi^{(3)}$ contribution in the phase-matched case

Qualitative analysis of the angular symmetry in the phase-matched experiment and comparison with the symmetry observed in the phase-mismatched experiments fails to provide evidence of $\chi^{(3)}$ or TFISH effects as the definitive source of the THz-driven SH modulation in BBO. However, higher order effects are always present, and additional insight can be gained by a more quantitative evaluation of the strength of these effects.

In the TFISH framework, the intensity of the SHG signal is proportional to three terms, a time-dependent heterodyne term, a time dependent homodyne term and a DC offset term:

$$I_{SHG}(t) \propto \left|\left(\chi^{(2)} + \chi^{(3)} E_{THz}(t)\right) E_{FF}^2\right|^2$$
$$\propto \underbrace{\left|\chi^{(2)} E_{FF}^2\right|^2}_{DC} + \underbrace{2Re(\chi^{(2)} \chi^{(3)} E_{THz}(t) E_{FF}^4)}_{Heterodyne \propto E_{THz}(t)} + \underbrace{\left|\chi^{(3)} E_{THz}(t) E_{FF}^2\right|^2}_{Homodyne \propto |E_{THz}(t)|^2} \quad \text{(S44)}$$

To link the intensity modulation to the optically driven phonons in the BBO, according to Ref.[18], $\chi^{(3)} E_{THz}(t)$ is identical to a time-dependent perturbation of the second-order non-linearity and can be rewritten in terms of the effective nonlinear coefficient d_{eff} and the phonon displacement Q:

$$\chi^{(3)} E_{THz}(t) = 2 \frac{\partial d_{eff}}{\partial Q} Q(t) \rightarrow \Delta d = \frac{\partial d_{eff}}{\partial Q} Q \quad \text{(S45)}$$

Therefore, the heterodyne contribution is proportional to the amplitude of the phonon, which is assumed proportional to the THz field.

DFT calculations of the phonon displacements can then be used to estimate the time-dependent 2nd-order nonlinear coefficients, Δd. Calculations of the phonon-amplitude dependent nonlinear coefficients are shown in Fig S6. By combining these coefficients with the phonon penetration depth δ_{ph} (calculated independently by DFPT), a simple two-layer model of the BBO crystal can be used to estimate the strength of the 3rd-order effects. This model consists of a uniformly pumped layer with a

constant phonon amplitude Q_0 that is δ_{ph} thick with the additional time-dependent nonlinearities, while the remainder of the crystal is considered to be at equilibrium.

The Type-I phase-matched component, which is proportional to $d_{22}^2 \left|E_y^{FF}\right|^4$ will be affected by the time-dependent term Δd_{22}, which is expected to have a maximum value of $\Delta d_{22} = 0.1\ pm/V$ with $d_{22} = 2.9\ pm/V$. In the 2-layer model which is expected to overestimate the strength of the effect, the relative 3rd-order intensity modulation of this component is calculated to be 0.68%, far below the modulation observed in the experiment. Furthermore, in terms of the angular dependence, this modulation will result in a uniform scaling of the original equilibrium 2-fold symmetric pattern, which is not observed in the experiment.

The strength of the Type-I/Type-II heterodyne component can also be calculated using the other time-dependent nonlinear components, $\Delta d_{25}, \Delta d_{26}$, and Δd_{35}. With the same 2-layer model, the relative intensity modulation that can be expected from this term is approximately 0.3%. While this value is again expected to be an overestimate, it is still far below the modulation observed in the experiment.

Again, the estimates of the strength of the $\chi^{(3)}$-effect fail to support a TFISH explanation of the intensity modulation in the phase-matched experiment.

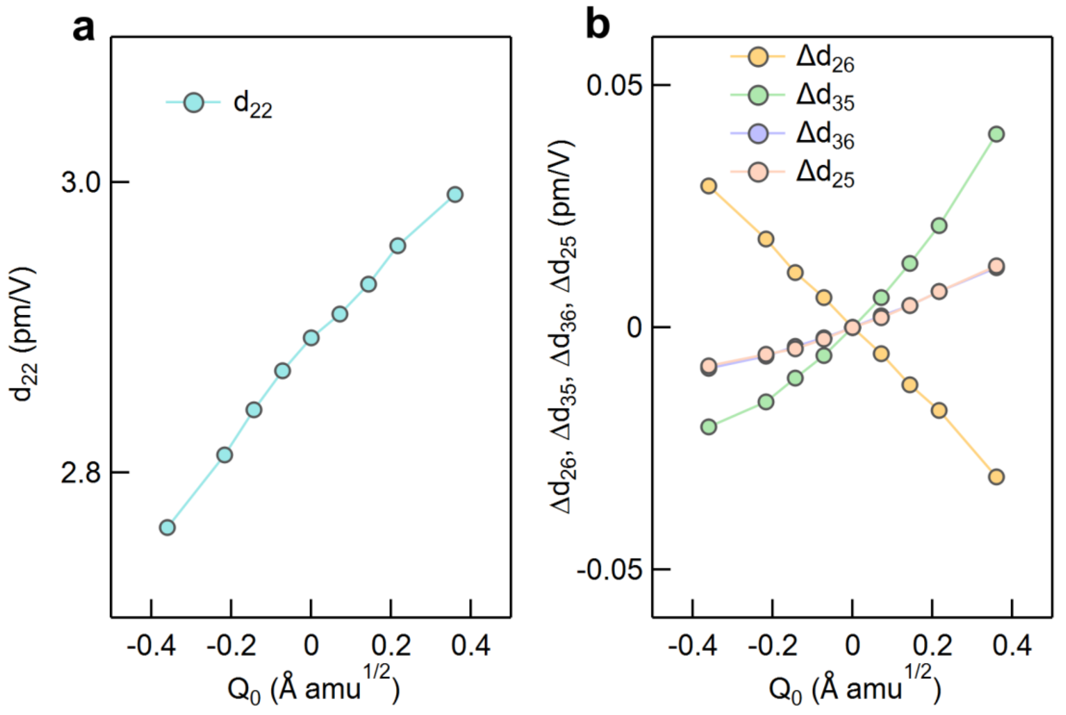

***Supplementary Fig. 6. a**, DFT calculated modulation of the nonlinear coefficients d_{22} as a function of lattice displacement Q_0. **b**, Modulation of the nonlinear coefficients Δd_{26}, Δd_{35}, Δd_{25}, and Δd_{36} as a function of Q_0. At the equilibrium structure, these coefficients are approximately zero.*

9. Estimation of the vibrational electro-optic coefficient and its frequency dependence

This section describes in detail the calculation of the vibrational electro-optic coefficient magnitude near the phonon resonance, as obtained from DFPT and from experimentally estimated values.

Calculation of the EO coefficient from DFPT: The modulation of the refractive index Δn is related to the phonon coordinate Q by:

$$\Delta n = \frac{n_x^3 r_{22} Q}{2}, \qquad \text{Eq. S46}$$

see Eq.2 in the main manuscript. Here, r_{22} denotes the phonon–optic coupling coefficient that quantifies the coupling between the phonon amplitude and the resulting refractive-index modulation.

From the DFTP results, Δn varies linearly with Q by which a value of $r_{22} = 0.0049\ \text{Å}^{-1}\ (amu)^{1/2}$ can be extrapolated from a linear fit, as discussed in the main text.

The standard vibrational EO coefficient, which is denoted here as r_{22}^E describes the variation of the index of refraction with respect to the applied electric field strength. This coefficient can be given in SI units (pm/V).

The conventional linear relationship between the phonon coordinate Q and the electric field E_{THz}: $Q(\omega) = \frac{\sqrt{\varepsilon_0 V}\omega_p}{(\omega_Q^2-\omega^2-i\Gamma\omega)} E_{THz}(\omega)$ is required to convert between the two coefficients. Substituting this relationship into Eq. S46 yields

$$\Delta n = \frac{n_x^3}{2} r_{22} \frac{\sqrt{\epsilon_0 V}\omega_p}{(\omega_Q^2 - \omega^2 - i\Gamma\omega)} E_{THz} = \frac{n_x^3}{2} \tilde{r}_{22}^E E_{THz} \qquad \text{Eq. S47}$$

Here, V is the volume of the unit cell, ϵ_0 is the vacuum permittivity, ω_Q, ω_p and Γ are the phonon frequency, phonon plasma frequency and the linewidth, respectively. Here, $\tilde{r}_{22}^E$ is the complex EO coefficient in SI units, and taking its real part:

$$r_{22}^E = Re\{\tilde{r}_{22}^{EO}\} = r_{22} \frac{\sqrt{\varepsilon_0 V}\omega_p(\omega_Q^2-\omega^2)}{\left(\omega_Q^2-\omega^2\right)^2 -(\Gamma\omega)^2} = r_{22}^E(0) \frac{(\omega_Q^2-\omega^2)}{\left(\omega_Q^2-\omega^2\right)^2 -(\Gamma\omega)^2}. \qquad \text{Eq. S48}$$

Note that Eq. S48 corresponds to the usual expression of the vibrational EO coefficient (see e.g. Ref. [19]), also shown in in Eq. S47.

Using $r_{22} = 0.0049\ \text{Å}^{-1}\ (amu)^{\frac{1}{2}}$ from the DFPT-derived refractive-index modulation, and $\omega_P = 6.57$ THz, $\omega_Q = 4.32$ THz and $\Gamma = 0.19$ THz determined by infrared spectroscopy, a maximum positive value of $r_{22-max}^E = 55 \frac{pm}{V}$ at $\omega_* = \sqrt{\omega_Q^2 - \Gamma\omega_Q} = 4.25$ THz is obtained. As discussed in the main text, the EO coefficient does not peak exactly at the phonon frequency ω_Q, the real part attains its maximum just below resonance at ω_* which is also consistent with the frequency peak of the SHG modulation response (see Fig. 5b of the main text).

A comparison of the calculated EO coefficient with experimental values can be performed in the DC limit. Extrapolating Eq. S48 to $\omega \to 0$ yields $r_{22}^E(0) = 6.6$pm/V, which is larger than the commonly reported experimental DC value of approximately 2.5 pm/V for BBO (Ref. [20]). This discrepancy likely reflects uncertainties in the experimental determination of the phonon plasma frequency, as well as the limitations of the single-phonon DFPT approach in accurately describing the low-frequency electro-optic response, which includes both electronic contributions and the cumulative effect of multiple phonon modes.

Experimental estimation of the EO coefficient from broadband THz pumping: Experimentally, the value of r_{22}^E can be estimated from the measured SHG modulation dynamics. As discussed in the main text, the relative SHG modulation, at fixed polarisation angle α is: $\Delta I_{rel}(t,\alpha) = \Delta I_{SH}(t,\alpha)/I_{SH,0}(\alpha)$,

where $I_{SH,0}(\alpha) = I_{SH,0}(0)\cos^4\alpha$. Measurements made at $\alpha = 30°$ were used to evaluate the EO coefficient.

Analytically, the modulation of the SH dynamics $\Delta I_{SH}(\alpha, t)$ in the time domain as a function of α (Eq. 2 in the main text) is:

$$\Delta I_{SH}(\alpha, t) = 4\, I_{SH,0}(0)\gamma(t)\frac{\Lambda(\delta_{ph}, L)}{L}\cos^3\alpha\sin\alpha, \quad \text{Eq. S49}$$

therefore,

$$\Delta I_{rel}(\alpha, t) = 4\,\gamma(t)\frac{\Lambda(\delta_{ph}, L)}{L}\frac{\sin\alpha}{\cos\alpha} = 4\kappa(\theta)r_{22}Q(t)\frac{\Lambda(\delta_{ph}, L)}{L}\tan\alpha. \quad \text{Eq. S50}$$

Here, $\kappa(\theta = 29.3°) = 65$ is the crystal geometrical factor, Λ is the parameter that accounts for the finite phonon penetration depth $\delta_{ph} = 30.2$ μm, within a crystal of thickness $L = 300$ μm and $Q(t)$ is the time dependent phonon coordinate. $Q(t)$ can be calculated using the phonon equation of motion leaving r_{22} as the only free parameter to find agreement with the experimentally measured relative modulation.

The phonon dynamics are described by:

$$\ddot{Q} + \Gamma\dot{Q} + \omega_Q^2 Q = \sqrt{\epsilon_0 V}\,\omega_p\tilde{E}_{THz}(t), \quad \text{Eq. S51}$$

where ϵ_0 is the vacuum permittivity, $V = 596$ Å^3 is the unit cell volume, and $\omega_Q = 4.32$ THz, $\omega_p = 6.57$ THz and $\Gamma = 0.19$ THz are the phonon frequency, phonon plasma frequency and the linewidth measured by THz-FTIR spectroscopy. $\tilde{E}_{THz}(t)$ is the THz field transmitted through the air-sample interface: $\tilde{E}_{THz}(t) = \frac{1}{2\pi\int\tilde{t}(\omega)E_{THz}(\omega)e^{-i\omega t}d\omega}$, where $\tilde{t}(\omega)$ is the complex Fresnel transmission calculated from the BBO refractive index (determined by THz FTIR), $E_{THz}(\omega)$ is the spectrum of the THz pump field measured by electro-optic sampling (EOS).

Solving Eq. S51 for $Q(t)$ and substituting into Eq. S50, agreement between the calculated relative modulation and measurement is obtained for a phonon-optic coupling coefficient, $r_{22} = 0.0130$ Å^{-1} (amu)$^{1/2}$ (Fig S7) with corresponding EO coefficient, $r^E_{22-max} = 145\ \frac{pm}{V}$.

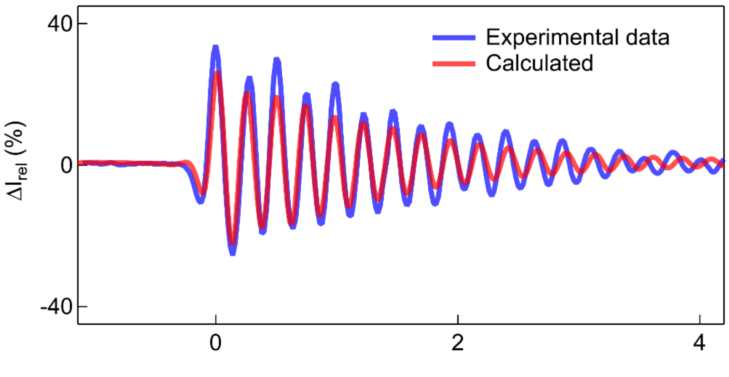

***Supplementary Fig.** 7. Experimentally measured ΔI_{rel} and calculated one using Eq. S50.*

Estimation of EO coefficient from narrowband THz pumping: Broadband THz pumping contains spectral components far from the phonon resonance which may interfere with the determination of r^E_{22}. Therefore, an important cross-check is to evaluate r^E_{22} with a narrowband THz excitation using band-pass THz filters to isolate spectral components centered around the phonon frequency.

The optical setup was based on a 1-kHz repetition-rate Ti:sapphire laser. THz radiation was generated in a DSTM crystal and driven by OPA signal pulses with an energy of 0.44 mJ per pulse. The resulting THz waveform and the corresponding field-strength estimation were obtained via EOS using a 200-µm-thick GaP crystal. The THz electric field was determined using the conventional expression (see, e.g., Ref.[21]), including the Fresnel transmission coefficient. The measured temporal waveform and its Fourier amplitude spectrum are shown in Supplementary Fig. 8a and Supplementary Fig. 8b, respectively.

The relative SH modulation dynamics $\Delta I_{\text{rel}}(\alpha, t)$, were recorded at the polarization angle ($\alpha = 30°$), and the corresponding Fourier amplitude spectrum is shown in Supplementary Fig. 8c and Supplementary Fig. 8d, respectively.

Following the same procedure as in the broadband THz case, the phonon optic coupling coefficient was found to be $r_{22} = 0.0160\ \text{Å}^{-1}\ (amu)^{1/2}$, which corresponds to $r^{E}_{22-max} = 178\ \frac{pm}{V}$, comparable to the value obtained using the broadband THz excitation.

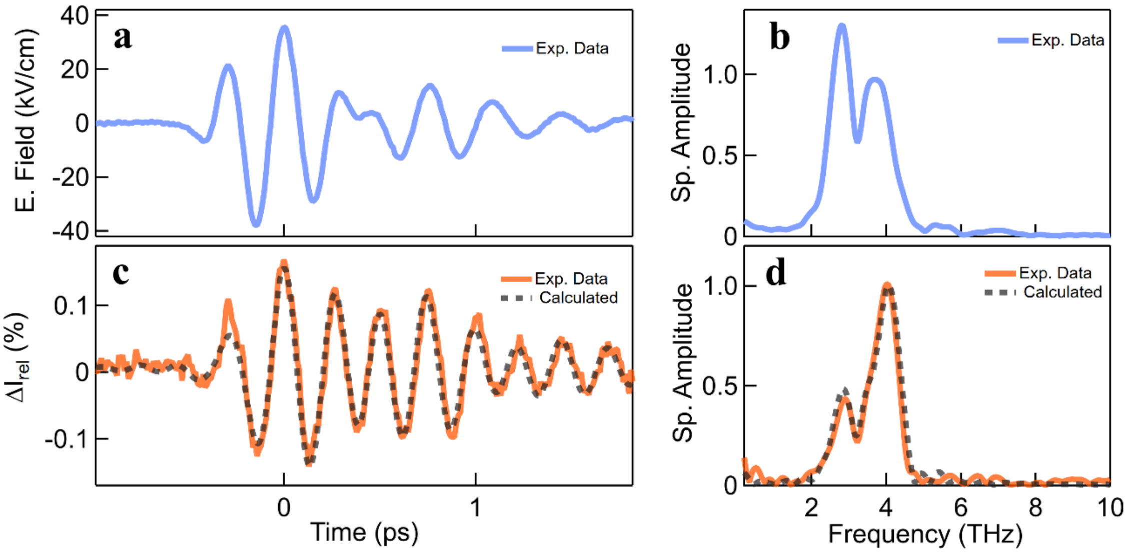

Supplementary Fig. 8. SHG modulation with a narrowband THz pulse. ***a***, *Time-domain THz pump waveform measured by EOS.* ***b***, *Amplitude spectra corresponding to* ***a***. ***c***, *THz-driven ΔI_{rel} and calculated one using Eq. S50.* ***d***, *Amplitude spectra corresponding to curves in* ***c***.

Supplementary References

1. Blanchard, F. *et al.* Improved terahertz two-color plasma sources pumped by high intensity laser beam. *Opt Express* 17, 6044–6052 (2009).

2. Valverde-Chávez, D. A. and Cooke, D. G. Multi-Cycle Terahertz Emission from β-Barium Borate. *J Infrared Millim Terahertz Waves* 38, 96–103 (2017).

3. Li, R. *et al.* Optical properties of barium borate crystal in the THz range revisited. *Opt Lett* 50, 686–689 (2025).

4. Liu, J., Guo, X., Dai, J. and Zhang, X.-C. Optical property of beta barium borate in terahertz region. *Appl Phys Lett* 93, 171102 (2008).

5. Kuzmenko, A. B. Kramers–Kronig constrained variational analysis of optical spectra. *Rev Sci Instrum* 76, 083108 (2005).

6. Ney, P., Fontana, M. D., Maillard, A. and Polgár, K. Assignment of the Raman lines in single crystal barium metaborate. *J Phys: Condens Matter* 10, 673–681 (1998).

7. Sutherland, R. L. *Handbook of Nonlinear Optics* (CRC Press, Boca Raton, 2003).

8. Yariv, A. Quantum Electronics (John Wiley & Sons, New York, 1988).

9. Li, R. Vibrational contributions to the electro-optic effect of BBO, KTP and RTP crystals. *Comput Mater Sci* 230, 112529 (2023).

10. Abarkan, M., Salvestrini, J. P., Fontana, M. D. and Aillerie, M. Frequency and wavelength dependences of electro-optic coefficients in inorganic crystals. *Appl Phys B* 76, 765–769 (2003).

11. Boyd, R. W. Nonlinear Optics (Third Edition) (Academic Press, Burlington, 2008).

12. Zhang, D., Kong, Y. and Zhang, J. Optical parametric properties of 532-nm-pumped beta-barium-borate near the infrared absorption edge. *Opt Commun* 184, 485–491 (2000).

13. Mankowsky, R., von Hoegen, A., Först, M. and Cavalleri, A. Ultrafast Reversal of the Ferroelectric Polarization. *Phys Rev Lett* 118, 197601 (2017).

14. Grishunin, K. A. *et al.* THz Electric Field-Induced Second Harmonic Generation in Inorganic Ferroelectric. *Sci Rep* 7, 687 (2017).

15. Bodrov, S. B., Sergeev, Yu. A., Korytin, A. I. and Stepanov, A. N. Terahertz-field-induced second optical harmonic generation from Si (111) surface. *Phys Rev B* 105, 035306 (2022).

16. Itoh, H. *et al.* Terahertz Field Control of Electronic-Ferroelectric Anisotropy at Room Temperature in $LuFe_2O_4$. *Phys Rev Lett* 135, 106504 (2025).

17. Li, C.-Y., Seletskiy, D. V., Yang, Z. and Sheik-Bahae, M. Broadband field-resolved terahertz detection via laser induced air plasma with controlled optical bias. *Opt Express* 23, 11436–11443 (2015).

18. Fang, Y. *et al.* Coherent manipulation of second-harmonic generation via terahertz-field mediated phonon-polariton in zinc oxide. *Nat Commun* 16, 5598 (2025).

19. Casalbuoni, S. *et al.* Numerical studies on the electro-optic detection of femtosecond electron bunches. *Phys Rev Accel Beams* 11, 072802 (2008).

20. Goodno, G. D. *et al.* Investigation of β-BaB_2O_4 as a *Q* switch for high power applications. *Appl Phys Lett* 66, 1575–1577 (1995).

21. Zhang, X.-C. and Xu, J. *Introduction to THz Wave Photonics* (Springer US, Boston, MA, 2010).
